# An eHealth and Health Literacy Educational Training to Support Older Patients With Type 2 Diabetes: Protocol for the JACARDI Interventional Study

**DOI:** 10.2196/78254

**Published:** 2025-12-24

**Authors:** Roberta Bevilacqua, Rachele Alessandra Marziali, Arianna Margaritini, Anna Rita Bonfigli, Elvira Maranesi, Elena Tortato, Maria Paola Luconi, Fabrizia Lattanzio, Cinzia Giammarchi

**Affiliations:** 1 IRCCS INRCA, Istituto di Ricovero e Cura a Carattere Scientifico - Istituto Nazionale di Riposo e Cura per Anziani, Via Santa Margherita 5, Ancona, 60124, Italy, 39 0718004767

**Keywords:** eHealth literacy, health literacy, older people, type II diabetes, educational training, protocol, pilot study

## Abstract

**Background:**

Type 2 diabetes is a prevalent chronic condition, particularly among older adults, and is associated with significant morbidity, mortality, and health care costs. Effective disease self-management is crucial for achieving glycemic control and preventing complications.

**Objective:**

This pilot study aims to evaluate the effectiveness of a structured, 5-week training program in improving eHealth literacy among older adults with type 2 diabetes and to assess its impact on health literacy, self-efficacy, psychological well-being, and clinical outcomes.

**Methods:**

In total, 200 community-dwelling adults aged ≥60 years with type 2 diabetes, a Mini-Mental State Examination score ≥24, a Geriatric Depression Scale score <9, and a low eHealth Literacy Scale score (≤29) will be recruited. Participants will attend five 90-minute weekly training sessions combining didactic and interactive modules focused on health literacy, diabetes self-management, and the use of digital tools. Assessments will be performed at baseline (T0), post intervention (T1), and at 1-month (T2) and 6-month (T3) follow-up. The primary outcome will be the change in eHealth literacy, as measured by the eHealth Literacy Scale, from baseline to post intervention. In addition, a wide range of secondary outcomes will be examined to capture the program’s multidimensional impact: improvements in general health literacy, self-efficacy in diabetes management, psychological well-being, and quality of life; reductions in diabetes-related emotional distress, body weight, and BMI; and better metabolic control, as measured by hemoglobin A_1c_ and lipid profiles. Finally, participants’ experiences, satisfaction, and perceived benefits will be explored to inform the future scalability and refinement of the program. A prestudy will be conducted, involving stakeholders, older adults, and caregivers, to refine the training content using co-design techniques. Descriptive statistics will be used to summarize sample characteristics. Preintervention and postintervention changes will be evaluated using 2-tailed paired *t* tests or their nonparametric equivalents, with repeated-measures models applied to follow-up data. A *P* value <.05 will indicate statistical significance.

**Results:**

The prestudy phase began in April 2025. Improvements in both health literacy and eHealth literacy are expected to be observed among participants, as assessed through validated instruments administered before and after the intervention.

**Conclusions:**

The Joint Action on Cardiovascular Diseases and Diabetes (JACARDI) study provides a structured and participatory approach to addressing the digital divide in chronic disease management among older adults. By focusing on personalized, evidence-based training, the study offers a promising model for empowering patients and improving health outcomes through enhanced digital and self-management competencies.

## Introduction

### Background

Diabetes is a chronic disease that affects people of all ages worldwide. It occurs when the pancreas does not produce enough insulin or when the body is unable to effectively use the insulin it produces. The former is known as type 1 diabetes, while the latter is known as type 2 diabetes. According to the World Health Organization (WHO), diabetes represents a global epidemic [1]. In fact, the number of people with diabetes has quadrupled since 1980 and is expected to continue growing. For example, in 2024, there were over 588 million individuals aged 20 to 79 years with diabetes worldwide, accounting for around 11% of all adults in this age group [2]. In Italy, slightly less than 5% of adults aged 18 to 69 years reported a diagnosis of diabetes in the biennium 2022‐2023; this percentage rose to almost 9% among individuals aged 50 to 69 years [3].

Given the widespread and growing prevalence of diabetes, it is essential to consider not only its health impact but also its broader complications and economic consequences. Beyond its health implications, the rising prevalence of diabetes imposes a significant economic burden on health care systems, accounting for around 12% of the total global health spending [2]. Due to their complexity and long-term treatment needs, complications resulting from poor diabetes management, including cardiovascular disease, kidney failure, and amputations, significantly increase the financial pressure on health systems [4].

Therefore, investing in effective prevention, education, and management strategies is a public health and economic priority [5].

Furthermore, while premature mortality due to other major noncommunicable diseases is decreasing, early deaths due to diabetes are increasing: there was a 5% increase between 2000 and 2016. Missed diagnosis or inadequate management of diabetes can lead to serious risks of debilitating and irreversible complications, including severe damage to the heart, eyes, kidneys, and nerves, which increases the risk of limb amputation, vision loss, and premature death [1].

One of the most important behavioral and therapeutic goals in the treatment of diabetes is glycemic control, which is a major aspect for reducing morbidity and mortality in people with type 2 diabetes (the most common type) [6].

At the core of diabetes management, glycemic control primarily involves patient self-management [7], which is achievable when a person with diabetes acquires the necessary knowledge (such as healthy nutrition, physical activity, blood glucose monitoring, and adherence to medication) and skills (eg, problem solving and healthy coping mechanisms) for behavior modification to better cope with the disease [8,9]. All these self-management behaviors fundamentally rely on health literacy as a foundational skill, as the ability to access, understand, and apply health information determines how effectively people with diabetes can engage in prevention, treatment adherence, and long-term disease control [10].

Indeed, people with lower health literacy levels tend to experience poorer overall health, higher rates of hospitalization and mortality, a lower ability to manage chronic diseases, and higher health care expenditures. In contrast, those with higher health literacy levels have a higher probability of using available health care services and making well-informed health care decisions [11]. Therefore, while a low level of health literacy may hinder the development of diabetes self-management skills [12], a high level is associated with confidence in diabetes self-management [13].

In this regard, studies have shown that interventions can improve glycemic control, quality of life, diabetic complications [8], and knowledge and skills of diabetes management [14].

However, despite these proven benefits, access to structured and effective self-management education remains uneven and inadequate in many cases, particularly among the most vulnerable populations with low socioeconomic status, limited education, language barriers, or limited access to health infrastructure [15]. These individuals often face additional difficulties in acquiring, understanding, and applying health information and, as a result, may be less likely to engage in sustained self-care behaviors, adhere to treatment regimens, or benefit from early prevention strategies. Therefore, addressing these gaps is essential to ensure that self-management interventions reach their full potential in improving population-level disease control.

However, digital exclusion can contribute to health inequalities and poorer health outcomes, particularly affecting older adults, marginalized groups, and people with disabilities, who have fewer opportunities to access or use digital health platforms. In response, 75% of WHO European Member States with digital health strategies have prioritized improving digital literacy and ensuring equitable access to fast, safe, and reliable internet [16]. Therefore, addressing eHealth literacy and digital inclusion is important in supporting effective diabetes self-management.

### Goal of the Study

This paper presents the protocol for the Joint Action on Cardiovascular Diseases and Diabetes (JACARDI) field trial, which aims to reduce the burden of cardiovascular disease and diabetes in European Union (EU) countries, at both the individual and societal levels. JACARDI is designed to integrate validated best practices and cost-effective interventions across countries and regions through transnational pilot initiatives, thereby complementing and reinforcing existing policies and programs. JACARDI will enhance cross-national collaboration, maximizing the exploitation of lessons learned through a clear strategy; engaging groups of interest; and promoting integration and sustainability of approaches to achieve high-level impact, including the implementation of effective interaction, cooperation, and cocreation between science and policy. In particular, this pilot study focuses on improving health literacy and awareness of cardiovascular disease and diabetes risks and risk factors, at both the individual and societal levels. It aims to reach people living with cardiovascular disease and diabetes and their care providers and to improve service pathways, self-management, and labor participation.

## Methods

### Field Trial

The JACARDI trial is a multicenter study coordinated by the Istituto di Ricovero e Cura a Carattere Scientifico-Istituto Nazionale di Riposo e Cura per Anziani (IRCCS INRCA) and conducted also at the Clinic of Endocrinology and Metabolic Diseases of the Azienda Ospedaliero-Universitaria delle Marche. Adopting a blended approach combining didactic and interactive training, the study primarily aims to improve eHealth literacy in older adults with type 2 diabetes, thereby promoting independent living and reducing the risk of health deterioration and impaired quality of life. The study consists of five phases: (1) recruitment and screening, (2) baseline assessment (T0), (3) a 10-week educational intervention, (4) postintervention assessment (T1), and (5) follow-up evaluations at 1 month (T2) and 6 months (T3).

Prior to the trial, a prestudy will be conducted to co-design and finalize the training content. A group of volunteers whose characteristics are similar to those of the target population will participate in focus groups to refine the training content and practical aspects prior to recruitment. The protocol was developed in accordance with the SPIRIT guidelines (Checklist 1).

**Figure 1.**
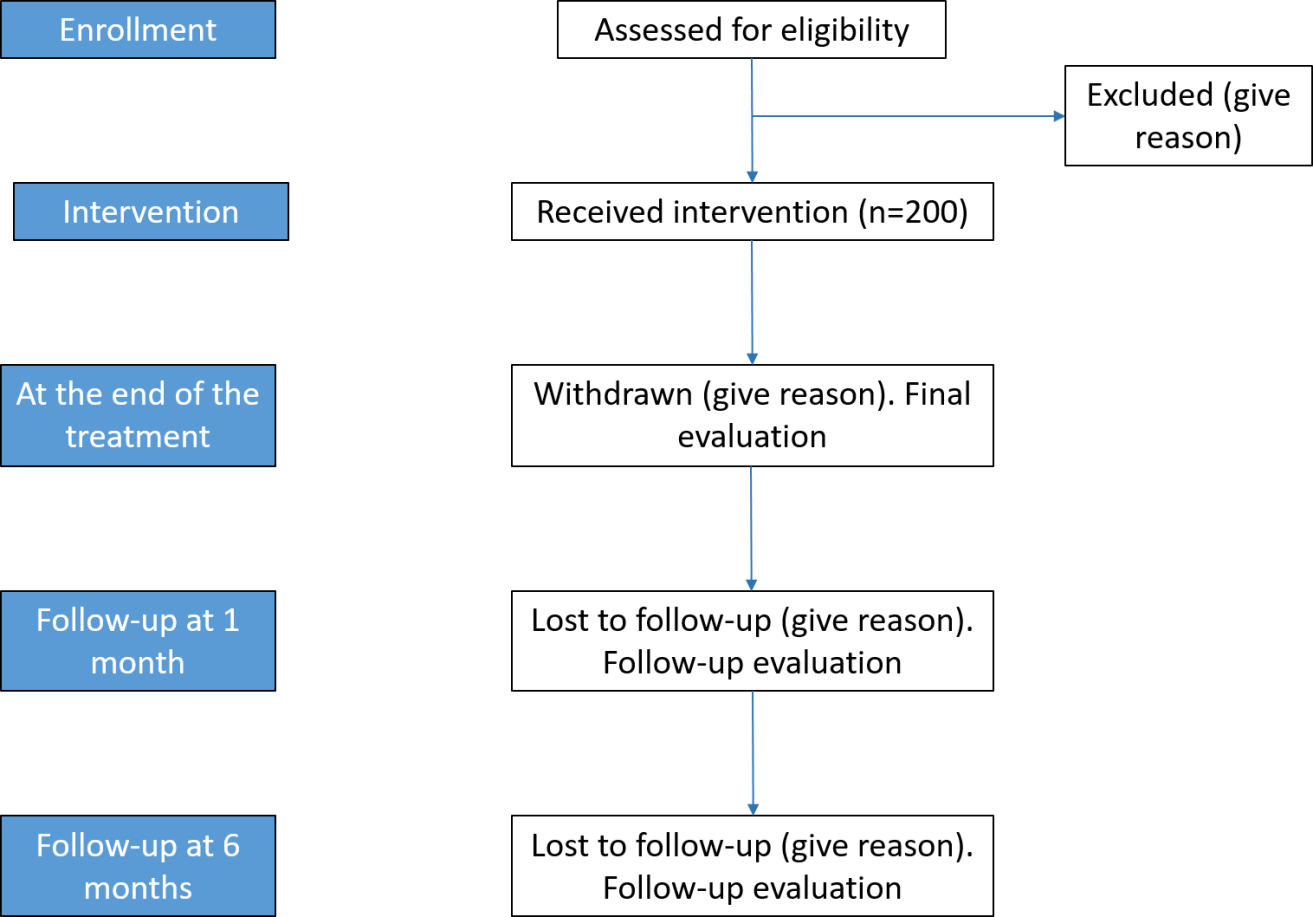
Flowchart of the study design, illustrating the screening process, the intervention and follow-up assessments, and the final sample analyzed.

Figure 1 shows the design of the JACARDI study.

### Prestudy Phase for Refinement of the Training Content

To evaluate the core concepts, strategies, and format for the pilot study, a preliminary prestudy will be conducted with a range of stakeholders, as outlined below:

1. Context analysis: A total of 10 semistructured interviews will be conducted with local experts (ie, service providers, regional and local policymakers, facility managers, and nongovernmental organizations) and health professionals (ie, medical doctors, nurses, psychologists, dietitians, and physiotherapists) to identify the enablers of and potential barriers to the pilot implementation, on the basis of the Consolidated Framework for Implementation Research (CFIR).
2. Focus groups: Two focus groups, each comprising 10 older adults, and one focus group consisting of 10 informal caregivers (not necessarily linked to the patients participating in the study) will be conducted to assess the validity of the proposed training concepts. Co-design techniques such as 6-3-5 brainwriting, simplified scoring, and open discussion will be used to refine the training content and practicalities.

The focus groups and interviews will be audio-recorded and subsequently transcribed in full. Then, the transcripts will be coded and analyzed using thematic analysis to identify key patterns and themes. Two researchers who will compare and discuss their findings to ensure consistency and credibility will collaboratively conduct the analysis and interpretation of results. Any discrepancies in interpretation will be resolved through discussion with a third senior researcher. The involvement of participants with a range of roles will contribute to the validity of findings. These procedures will enhance the rigor and trustworthiness of the co-design phase.

Indeed, the results of the prestudy phase will facilitate the refinement of the training sessions’ core content, which will be assessed through the pilot intervention. Specifically, the content, format, and delivery methods of the intervention will be adapted according to the themes that emerge from the focus groups and interviews. This will ensure that the final program reflects the needs, preferences, and lived experiences of the target population and stakeholders. The inclusion and exclusion criteria set for the JACARDI trial will be adopted in the prestudy phase as well, with the objective of involving older patients who exhibit the same characteristics as those in the final trial. Regarding caregivers and other stakeholders such as policymakers, there are no specific criteria in place other than being a carer of a diabetic person and working in the health policy sector, respectively.

### Participant Recruitment

The recruitment will be conducted in the selected centers under the coordination of the IRCCS INRCA. Following a 2-week reflection period, individuals who confirm their wish to participate in the research in writing or verbally, after having read the information letter and the consent form received by email, will be invited by the investigator to consult a doctor in the 3 months preceding the start of the research. The doctor volunteering to carry out the inclusion visits will first provide the participant with the information letter and 2 consent forms. After the participant and the doctor carefully read and sign the consent forms, the doctor will use anamnesis, the Mini-Mental State Examination (MMSE), the Geriatric Depression Scale (GDS, 5-item version), and the eHealth Literacy Scale (eHEALS) to ascertain the participant’s eligibility for the research study. This medical examination will take place before the study begins. Participants who sign the consent form but for whom the fulfillment of the inclusion criteria is not confirmed by the doctor will not be included in the research.

To ensure participants’ ability to engage actively in the training activities and complete the assessments, certain criteria were applied to assess cognitive health (MMSE score ≥24) and physical health (eg, absence of severe mobility limitations, advanced neurodegenerative diseases, or significant sensory impairments). These requirements are intended to minimize participant burden, ensure safety during in-person group sessions, and reduce attrition due to difficulties in attending sessions or completing questionnaires. While this approach improves the internal validity and feasibility of the pilot implementation, it may limit the representativeness of the sample with respect to the broader population of older adults with type 2 diabetes. The inclusion and exclusion criteria are reported in Textbox 1.

Textbox 1. **Inclusion and exclusion criteria for patients.**

**Inclusion criteria**

- Age ≥60 years
- Capacity to consent
- With type 2 diabetes
- eHealth Literacy Scale (eHEALS) score ≤29 (validated cutoff for low eHealth literacy [17])
- No acute or untreated medical problems
- Mini-Mental State Examination (MMSE) score ≥24 (validated cutoff for cognitive function [18])
- Geriatric Depression Scale (GDS) score <2 (validated cutoff for depressive symptoms [19])

**Exclusion criteria**

- Failure to meet the inclusion criteria
- Simultaneous participation in other studies
- Lack of written informed consent
- History of a myocardial infarction or stroke within the past 6 months
- Painful arthritis, spinal stenosis, amputation, painful foot lesions, or neuropathy, limiting balance and mobility
- Uncontrolled hypertension
- Advanced Parkinson disease or other neuromuscular disorder
- Diagnosis of metastatic cancer or treatment with immunosuppressive therapy
- Significant visual or hearing impairment

Following the medical visit, the investigator will contact the potential participant by email or telephone to discuss the study and confirm their interest in participation. At this stage, detailed information about the study procedures will be provided, and written informed consent will be obtained before any study-related activities are initiated. The schedule for the visits and the procedures for conducting the research at the participant’s home will then be agreed upon according to participant availability. When recruitment is complete, participants included in the study will undergo all routine clinical investigations, such as collection of blood samples to assess their glycemic and lipid profiles and measurement of BMI. In addition, a researcher will perform the preliminary evaluation (T0). The scales described in Textbox 2 will be administered, and then an interview will be conducted. The participant will undergo training sessions at the study centers with the other study participants. After 10 weeks, the final evaluation will be conducted (T1). Participants will be required to complete a series of questionnaires, the details of which can be found in Table 1. During this phase, blood sample collection and BMI measurement will not be performed.

During the 2 follow-up visits (1 month and 6 months following the conclusion of the intervention), any alterations in the patient’s medical history will be recorded. All routine clinical investigations and all planned assessment scales will be administered. Blood sample collection to assess the glycemic and lipid profiles and BMI measurement will be performed exclusively at T3.

Textbox 2. **Outcomes of the study.**

**Primary outcomes**

- Improvement in eHealth literacy, assessed using the eHealth Literacy Scale (eHEALS) [17]

**Secondary outcomes**

- Improvement in self-efficacy, evaluated using the Diabetes Management Self-Efficacy Scale (DMSES) [20]
- Reduction in emotional distress related to diabetes, measured using the Problem Areas in Diabetes Questionnaire (PAID) [21]
- Improvement in psychological well-being, assessed using the Psychological Well-Being Scale (PWB) [22]
- Improvement in quality of life (QoL), assessed using the EQ-5D-5L Visual Analog Scale (VAS) [23] and 12-item Short Form Health Survey (SF-12) [24]
- Reduction in body weight (measure routinely collected at the Diabetology Unit)
- Reduction in BMI (measure routinely collected at the Diabetology Unit)
- Improvement in the glycemic profile (glycated hemoglobin; measure routinely collected at the Diabetology Unit)
- Improvement in the lipid profile (directly related to cardiovascular risk; measure routinely collected at the Diabetology Unit)
- Collection of information about the participants’ experience with the training, their level of satisfaction, any benefits they have experienced, and any improvement suggestions, using an ad hoc questionnaire
- Improvement in health literacy, assessed using the 16-item European Health Literacy Survey Questionnaire (HLS-EU-Q16) [25]

**Table 1.** Tools and dimensions of the protocol.

Dimension	Tool	R^a^	T0^b^	T1^c^	T2^d^	T3^e^
Cognitive status	MMSE^f^	X	—^g^	—	—	—
Psychological status	GDS^h^ 5-item version	X	—	—	—	—
General information	Sociodemographic questionnaire	X	—	—	—	—
Quality of life	EQ-5D-5L VAS^i^, SF-12^j^	—	X	X	X	X
Psychological well-being	PWB^k^ (18 items)	—	X	X	X	X
eHealth literacy	eHEALS^l^	X	—	X	X	X
Health literacy	HLS-EU-Q16^m^	—	X	X	X	X
Diabetes-related emotional distress	PAID^n^	—	X	X	X	X
Self-efficacy	DMSES^o^	—	X	X	X	X
Training experience	Ad hoc questionnaire	—	—	—	—	X

a R: recruitment.

b T0: first evaluation.

c T1: final evaluation.

d T2: follow-up 1 month after the end of the training.

e T3: follow-up 3 months after the end of the training.

f MMSE: Mini-Mental State Examination.

g not applicable

h GDS: Geriatric Depression Scale.

i VAS: Visual Analog Scale.

j SF-12: 12-item Short Form Health Survey.

k PWB: Psychological Well-Being Scale.

l eHEALS: eHealth Literacy Scale.

m HLS-EU-Q16: 16-item European Health Literacy Survey Questionnaire.

n PAID: Problem Areas in Diabetes Questionnaire.

o DMSES: Diabetes Management Self-Efficacy Scale.

### Intervention Through Training

The training is structured as a blended program combining didactic and interactive educational methods, designed to strengthen health and eHealth literacy and to support diabetes self-management in older adults. The program is organized into 5 modules over a 10-week period (1 session every other week). The average duration of each session will be 90 minutes. Active engagement will be encouraged throughout the sessions, with participants involved in discussions, scenario-based activities, and guided reflection. This approach aims not only to promote meaningful learning but also to foster confidence, autonomy, and sustainable behavioral change in managing type 2 diabetes. The overarching objectives of the training program are grounded in the three key learning competencies outlined by the European Qualifications Framework, which are articulated through the following three dimensions:

1. Knowledge: The program aims to cultivate awareness and critical understanding regarding health and eHealth literacy. The objective is to empower participants with comprehensive knowledge of health-related issues and eHealth literacy challenges. By delving into these subjects, participants will grasp their significance and explore effective strategies for addressing them.
2. Skills: The training is designed to equip older adults with advanced skills that are specifically tailored to their interactions with digital tools in the context of health and eHealth literacy. Through personalized instruction, participants will be equipped with the necessary capabilities to navigate digital platforms effectively, thereby fostering proficiency in the use of these tools to enhance health literacy among older adults.
3. Competencies: The program is committed to ensuring the sustainability of the skills acquired during the training and their practical application in real-world scenarios. Beyond mere knowledge acquisition, the objective is to catalyze meaningful behavior change. The training environment is designed to encourage the adoption, adaptation, and maintenance of positive behavioral habits, thereby addressing health and eHealth literacy challenges comprehensively.

The training program is divided into the following modules:

1. Module I: raising awareness of eHealth and health literacy: introduction to the aims and context of JACARDI and diabetes
2. Module II: achieving new knowledge: health literacy and self-management of diabetes
3. Module III: achieving new knowledge: technological and nontechnological strategies to support the self-management of diabetes
4. Module IV: practicing new skills: usability with practice session
5. Module V: self-evaluation and sustainability of the improvement (final questionnaire)

Module I aims to enhance participants’ awareness of the importance of eHealth and health literacy, highlighting their crucial role in managing type 2 diabetes among older adults. To this end, the module is divided into two distinct parts: a lecture session and a group discussion. The first part focuses on analyzing the definition and relevance of eHealth and health literacy, highlighting the difficulties older adults may encounter in adopting digital tools for health care. An overview of the Italian initiatives and projects that focus on the digitization of health care services for older adults is also provided. Particular attention is paid to the obstacles to implementation, such as regulatory issues, privacy concerns, economic sustainability, and challenges posed by multimorbidity. The use of practical examples and short demonstrations facilitates the understanding of the theoretical concepts. The second part of the module consists of a group discussion, during which participants will be invited to reflect on and share their opinions on the role of technology in health care. This part of the module employs guided discussion techniques and real-world scenarios to encourage dialogue on the acceptance or rejection of technological tools and to identify the main barriers to access. The barriers include motivational limitations, deficiencies in digital skills, material constraints, and usability challenges. This configuration facilitates the learning process, combining a solid theoretical foundation and practical activities, emphasizing the direct correlation between learning objectives and final outcomes.

Module II aims to address issues related to improving self-management and health literacy among older adults with type 2 diabetes, foster a greater understanding of the disease, and support the adoption of effective strategies for the daily management of type 2 diabetes. The session is structured around interactive lessons that offer participants the opportunity to learn fundamental information about the disease and prevention of its negative consequences. During the first part, definitions and guidelines will be shared on key topics such as treatment management, importance of physical activity, adoption of a personalized nutritional plan, and diabetic foot care. The goal of this phase is to reduce inadequate health literacy, which is often associated with difficulties in understanding medical information, limited knowledge of the disease, poor adherence to treatment, worsening health, and increased costs. Classes will include hands-on demonstrations and the use of concrete examples to facilitate learning and make complex medical concepts more accessible. The second part of the session is structured as a group discussion moderated by a clinical psychologist. In this context, participants will have the opportunity to share and discuss the most common obstacles and fears encountered in the diabetes management journey as well as to discuss concerns about the consequences of the disease and the impact of the diagnosis on their lives. The methodology adopted, which is based on real-world scenarios and direct comparison, helps foster behavioral change, sustain motivation, and promote practical strategies for successfully addressing everyday challenges.

Module III is designed to assist older patients with type 2 diabetes in acquiring and understanding the concepts of health literacy and eHealth literacy. The objective is to support them to make more informed health decisions and use digital tools effectively for their physical and mental well-being. The module combines a theoretical component with a practical experience. Initially, the key concepts, the available resources, and the relevant associations and stakeholders, including peer support groups, will be presented and discussed. The goal is to demonstrate to the participants the range of technological and nontechnological strategies that are effective in enhancing eHealth skills and facilitating diabetes management. The teaching methods adopted include interactive presentations, guided discussions, and hands-on activities. The digital applications selected for the program are chosen on the basis of a preliminary study that evaluated their ease of use and reliability using focus groups and interviews. During the practical session, participants will engage in exercises designed to select safe and intuitive digital apps, with the aim of promoting a more autonomous and informed approach to managing their health. The learning-by-doing methodology will enable participants to directly test practical tools, verify their functionality, and gain greater confidence in using digital technologies, integrating these skills into their daily diabetes management. This approach facilitates the integration of digital solutions into participants’ daily routines, optimizing health management practices and fostering the development of skills useful for greater autonomy and decision-making capacity.

Module IV explores the pivotal concept of usability in the context of digital and technological devices and services, with the aim of raising awareness of interaction with technology among older patients with type 2 diabetes. The session is designed as a lecture, with the objective of conveying to participants the idea that technology is not inherently good or bad in itself. Rather, shortcomings in the usability of tools are often the main cause of frustration and, consequently, rejection. The module focuses on the most common difficulties and errors in using technology, assisting participants in distinguishing between issues stemming from insufficient digital skills and limitations related to the usability of a tool or service. The methodology combines theoretical explanations and practical activities. In the final part of the module, participants will engage in a practical usability test, with a focus on frequently used digital platforms, such as those employed for scheduling medical appointments, which are particularly relevant to the health care needs of older people. This exercise enables participants to directly experience the main usability issues encountered on these platforms, addressing the difficulties that can arise in everyday life. Through a problem-solving approach, participants will identify the most common obstacles in interacting with technology and acquire strategies to overcome them, thus improving their independence and effectiveness in using digital services. By actively participating in the hands-on exercise, patients will refine their abilities in navigating digital platforms, thereby fostering a more informed and constructive interaction with technology. This contributes not only to more independent management of digital services but also to improved health care and monitoring.

Module V is designed to encourage participants to self-reflect, with the objective of evaluating their level of awareness, knowledge, and skills in technology and digital health literacy. This session represents the final stage of the training program, including a component dedicated to evaluating the effectiveness of the educational activities and methods used during the program. The method adopted involves a trainer guiding participants through the completion of the final questionnaire, explaining its characteristics and providing practical support during the completion. During this phase, participants will complete a series of validated instruments, described in detail in the Results section of the protocol, which will measure changes in quality of life, psychological well-being, health literacy, eHealth literacy, self-efficacy, and diabetes-related distress. The use of standardized scales ensures that the assessment is measurable and comparable across multiple time points, thereby providing clear indicators of the program’s effectiveness. This quantitative evaluation is complemented by a final discussion, in which participants’ reflections, feedback, and suggestions for maintaining the skills acquired will be collected. Consequently, the evaluation process evolves from a mere descriptive exercise to one that is also actionable, as the collected data will be systematically analyzed and used to identify strengths and areas for improvement, guiding the refinement of future training cycles. The module includes instructions for completing the questionnaire, reflecting on personal progress in relation to the training objectives, and receiving constructive feedback. By actively participating in this self-assessment process, participants will have the opportunity to strengthen their digital and health literacy skills, while trainers will use the information gathered to optimize future training initiatives and ensure continuous improvement of digital health literacy interventions. In this manner, the program combines theory and practical application to foster ongoing change and hold participants accountable for their learning journey.

The training sessions will be facilitated by a multidisciplinary team of health care professionals and educators with expertise in diabetes management, health literacy, and digital health. The trainers will generally include doctors, nurses, dietitians, and clinical psychologists, each with previous experience in patient education and group facilitation. At each center, depending on the staff present and the topic of the session, the facilitators may vary.

To ensure consistency and fidelity across sessions and locations, the coordination center will be responsible for the preparation of all training materials, including manuals, slides, and practical exercises. These materials will be disseminated to all locations before the program begins. In addition, online and in-person familiarization sessions will be conducted for all trainers to review content, clarify any questions, and practice delivering the modules. This will ensure that all trainers are familiar with the course materials. Trainers will also receive guidance on specific strategies for addressing particular issues, such as managing emotional challenges or promoting autonomy, to maintain a supportive approach throughout the program. These sessions are designed to ensure that training is conducted in the same way across all centers, while still allowing trainers to adapt to the needs of participants.

### Outcomes

The primary end point of the study is defined as an improvement in eHealth literacy following the implementation of the training program, scheduled to span a period of 10 weeks, as described in Textbox 2. Then, the field trials will focus on improvements in health literacy, self-efficacy, psychological well-being, and quality of life, in addition to glycemic and lipid profiles. Moreover, the study aims to minimize emotional distress and reduce body weight and BMI, as shown in Textbox 2.

For these reasons, the protocol includes study-specific questions on demographics, emotional distress, psychological well-being, and physiological aspects. All scales used in the study have been validated in the languages of the pilot sites and are deemed suitable for administration to the patients enrolled in the study. Data will be collected at baseline (T0), immediately post intervention (T1), and at the 1-month (T2) and 6-month (T3) follow-up visits, as detailed in Table 1.

### Statistical Analysis

The eHEALS score was used for sample size calculation. Specifically, the sample size was determined using the combination of a pilot study [26] and a larger study [27].

In the first case, considering a 1-tailed *t* test for paired samples in which the primary outcome eHEALS score varies from a mean of 24.3 (SD 8.9) to 28.4 (SD 8.1) [26], corresponding to an effect size of 0.48, an α error of .05 and a power of 90%, the sample size should be 39 participants. Considering a dropout rate of 15%, the sample size increases to 45 participants. While larger effect sizes reported in the literature [27] would suggest even smaller sample sizes, we decided to include 200 participants for several reasons: (1) to ensure robust and generalizable results across a diverse population of older adults with type 2 diabetes, (2) to account for potential variability in baseline eHealth literacy and other characteristics, (3) to maintain statistical power for secondary outcomes and subgroup analyses, and (4) to address existing gaps in the literature by providing sufficiently powered evidence on the efficacy of the intervention.

The initial phase of the data analysis will entail the description of the sample. Continuous variables will be reported as either the mean and SD or the median and IQR on the basis of their distribution (assessed using Kolmogorov-Smirnov test). Categorical variables will be expressed as an absolute number and percentage. In the context of group comparisons, continuous variables will be compared using independent or 2-tailed paired *t* tests for normally distributed data and Mann-Whitney *U* or Wilcoxon signed-rank tests for nonnormally distributed data, whereas categorical variables will be compared using chi-square or Fisher exact tests, as appropriate. A significance level of *P*<.05 will be applied. Corrections for multiple comparisons (eg, the Bonferroni or Holm method) will be applied where necessary. The results will be compared with the data from the other partner countries. Descriptive statistical analyses will be performed on the quantitative data with SPSS software (version 29.0.1.0; IBM Corp) or Rstudio (version 4.4.1; Posit PBC).

### Ethical Considerations

The Marche Territorial Ethics Committee (CET MARCHE) approved the study on June 27, 2024 (Prot. INRCA 23332). The study protocol was registered on ClinicalTrials.gov (NCT06904690).

The study adheres to the principles of the Declaration of Helsinki and Good Clinical Practice guidelines [28,29]. All participants in this study will be required to provide written informed consent. Personal data collected during the trial will be handled and stored in accordance with the General Data Protection Regulation (GDPR) 2018 [30]. To this end, data will be anonymized at the end of the study. The use of the study data will be controlled by the principal investigator. All data and documentation related to the trial will be stored in accordance with applicable regulatory requirements, and access to the data will be restricted to authorized study personnel. The consent form includes detailed information on study procedures, risks and benefits, data protection and storage, right to withdraw or request erasure of personal data, and the possibility of data being used in anonymized form for future research, in line with the EU Open Research Data guidelines.

### Data Management and Protection

Personal identifiers will be stored separately from the research data and pseudonymized at the time of collection. The pseudonymized datasets will be stored on secure institutional servers with password-protected access restricted to authorized study personnel only. Screening data will be discarded at the conclusion of the project, and research data will be anonymized and made openly available for secondary analyses 3 years after project completion, in accordance with the EU Open Research Data requirements. Participants will be informed of their rights to access, correct, withdraw, or erase their data at any time.

Study oversight will be provided by the principal investigator and the ethics committee. Participants will be monitored throughout the intervention period, and any adverse events or protocol deviations will be documented and reported to the ethics committee according to institutional procedures.

## Results

The recruitment process is scheduled to start in September 2025 and will continue until September 2026. The prestudy phase, aimed at evaluating the core concepts, strategies, and format designed for conducting the pilot study and refining the training content, began in April 2025. The rating scales used are shown in Table 1, grouped by study phase. Improvements in both health literacy and eHealth literacy are expected to be observed among participants, as assessed through validated instruments administered before and after the intervention. Enhancements in diabetes self-management behaviors, such as medication adherence, dietary habits, and glucose monitoring, are also anticipated. Moreover, an increased level of literacy is expected to result in greater self-efficacy and confidence in the use of digital health tools. Positive changes in psychosocial outcomes, including quality of life, perceived autonomy, and reduction in diabetes-related distress, are also expected. These outcomes will be systematically evaluated as part of the JACARDI study, providing comprehensive evidence on the effectiveness of the intervention and its impact on the health literacy, self-management, and psychosocial well-being of older adults.

## Discussion

The JACARDI pilot study addresses a critical public health concern, namely the management of type 2 diabetes in older adults, through the enhancement of eHealth and health literacy. The study design integrates a robust evidence-based rationale with a comprehensive, blended educational intervention that is innovative both in its methodology and in its participatory, patient-centered approach.

By focusing on older adults with low eHealth literacy, the project targets a particularly vulnerable population often excluded from digital health innovations due to age-related barriers, cognitive limitations, and digital divides. it is an established fact that older adults with low eHealth literacy often encounter significant barriers such as unfamiliarity with technology, limited access to devices or internet, and low confidence in navigating digital platforms. This population is frequently underrepresented in digital health initiatives, despite bearing a significant disease burden and having the greatest potential to benefit from accessible, supportive interventions. Addressing these disparities is crucial for ensuring that technological advances in health care do not widen existing health inequities. The modular approach allows for the gradual development of knowledge, skills, and behavioral competencies required for the autonomous management of chronic conditions.

The inclusion of a prestudy phase involving stakeholders and potential beneficiaries allows for co-design and adjustment of the training materials, thereby increasing the relevance, usability, and acceptability of the intervention. Additionally, the training content includes not only theoretical and technical skills but also emotional and motivational components that are critical for sustaining long-term behavioral change. The modules encompass not only disease-specific knowledge but also stress management strategies and coping techniques to enhance behavioral change. Special attention has been paid to promoting self-efficacy, with activities designed to increase self-confidence and reduce anxiety when using digital tools.

From a methodological point of view, the study adopts a robust evaluation framework using validated tools and multiple follow-up time points, thereby enabling both short-term and midterm outcome monitoring. The use of both subjective (eg, questionnaires) and objective (eg, glycemic and lipid profiles) measures allows for a multidimensional evaluation of the intervention’s impact.

However, it is important to acknowledge some limitations. This being a pilot study, the generalizability of the results may be constrained by the specific characteristics of the local settings and participants. Moreover, despite efforts made to personalize the intervention, its scalability across different health care systems and sociocultural environments may require further adaptations. Potential barriers include disparities in digital access or digital literacy among older adults, as well as local infrastructure constraints that may affect intervention delivery. To address these issues, the study will employ a blended training format combining digital and in-person components and provides technical support and guidance to participants. Feedback will be collected to inform future adaptations for broader implementation. In addition, the exclusion criteria—particularly the exclusion of individuals with severe mobility limitations, advanced Parkinson disease, and significant visual or hearing impairment—may restrict the external validity of the findings. The establishment of these criteria was deemed necessary to ensure the attendance of all participants to the requisite training sessions and to facilitate their effective engagement with the digital tools that will be introduced. Nevertheless, they also exclude subgroups that may be among the most vulnerable to poor digital health engagement. It is recommended that future studies consider tailored strategies (eg, home-based or caregiver-assisted training, accessible digital interfaces) to reach these populations and evaluate whether adapted interventions can address their specific needs.

Despite these challenges, the JACARDI pilot study represents a valuable contribution to the growing field of digital health literacy and chronic disease self-management.

The JACARDI pilot project is designed to provide valuable insights for future large-scale interventions aimed at reducing health inequalities among older adults with type 2 diabetes. The integration of digital health literacy into chronic disease management has the potential to foster greater patient accountability, improve self-care practices, and contribute to reducing the burden on health care systems. Should this approach prove effective, it could be replicated and adapted to different settings, thereby supporting ongoing efforts to transform health literacy principles into patient-focused interventions. Further research is required to assess the long-term sustainability, clinical effectiveness, and cost-effectiveness of these programs, as well as their potential introduction into routine care.

## Supplementary material

10.2196/78254 Checklist 1 SPIRIT checklist.

## Abbreviations

CFIR: Consolidated Framework for Implementation Research
eHEALS: eHealth Literacy Scale
EU: European Union
GDPR: General Data Protection Regulation
GDS: Geriatric Depression Scale
IRCCS INRCA: Istituto di Ricovero e Cura a Carattere Scientifico - Istituto Nazionale di Riposo e Cura per Anziani
JACARDI: Joint Action on Cardiovascular Diseases and Diabetes
MARCHE: Marche Territorial Ethics Committee
MMSE: Mini-Mental State Examination
SPIRIT: Standard Protocol Items: Recommendations for Interventional Trials
T0: first evaluation
T1: final evaluation
T2: follow-up 1 month after the end of the training
T3: follow-up 3 months after the end of the training
WHO: World Health Organization
